# A Cohort Study of Liver Involvement in Patients With Adult-Onset Still's Disease: Prevalence, Characteristics and Impact on Prognosis

**DOI:** 10.3389/fmed.2020.621005

**Published:** 2020-12-23

**Authors:** Huihui Chi, Zhihong Wang, Jianfen Meng, Pingyang Han, Limin Zhai, Tienan Feng, Jialin Teng, Yue Sun, Qiongyi Hu, Hao Zhang, Honglei Liu, Xiaobing Cheng, Junna Ye, Hui Shi, Xinyao Wu, Zhuochao Zhou, Jinchao Jia, Liyan Wan, Tingting Liu, Xin Qiao, Mengyan Wang, Fan Wang, Xia Chen, Chengde Yang, Yutong Su

**Affiliations:** ^1^Department of Rheumatology and Immunology, Ruijin Hospital, Shanghai Jiao Tong University School of Medicine, Shanghai, China; ^2^Department of Rheumatology and Immunology, The First People's Hospital of Yancheng, The Forth Affiliated Hospital of Nantong University, Yancheng, China; ^3^Department of Rheumatology and Immunology, The Affiliated Hospital of Hangzhou Normal University, Hangzhou, Zhejiang, China; ^4^Department of Endocrinology, The Second Affiliated Hospital of Anhui Medical University, Hefei, China; ^5^Shanghai Tongren Hospital/Clinical Research Institute, Hongqiao International Institute of Medicine, Shanghai Jiao Tong University School of Medicine, Shanghai, China; ^6^Shanghai Clinical Research Promotion and Development Center, Shanghai Shenkang Hospital Development Center, Shanghai, China; ^7^The Second Affiliated Hospital of Chengdu Medical College, China National Nuclear Corporation 416 Hospital, Chengdu, China

**Keywords:** adult-onset Still's disease, liver involvement, refractory, treatment, prognosis

## Abstract

**Objective:** Adult-onset Still's disease (AOSD) is a systemic disorder commonly accompanied by liver involvement. This study aims to illustrate the detailed information of liver abnormalities in patients with AOSD and evaluate the impact on the prognosis.

**Methods:** A total number of 128 hospitalized patients, who met the Yamaguchi criteria of AOSD in the Department of Rheumatology and Immunology, Ruijin Hospital from July 2016 to August 2019 were consecutively enrolled and followed up. The demographic characteristics, clinical features, laboratory tests, treatments and prognosis were recorded. Correlations of liver function tests (LFTs) with disease activity and laboratory parameters were analyzed by the Spearman test. Risk factors of the refractory AOSD were evaluated by multivariate logistic regression analysis.

**Results:** Liver involvement was presented in 104 (81.3%) patients with AOSD. We observed that 34 (32.7%) patients were with mild elevation, 32 (30.8%) patients were with moderate elevation, and 38 (36.5%) patients were with severe elevation. The majority of elevated ALT, AST and ALP decreased to normal within the range of 2 months, except for GGT. Furthermore, the LFTs were found significantly correlated with disease activity. Besides, we found patients with higher levels of LFTs tended to require more intensive treatments and suffered from poorer prognosis. Multivariate logistic regression analysis showed ALP ≥ 141 IU/L and GGT ≥ 132 IU/L are independent risk factors of refractory AOSD.

**Conclusion:** Liver involvement is common in patients with AOSD, the levels of LFTs are associated with disease activity and related to the treatment strategies and prognosis.

## Introduction

Adult-onset Still's disease (AOSD) is a rare systemic autoinflammatory disorder. The etiology and pathogenesis of AOSD still mostly undetermined (1, 2). Patients with AOSD often presented with high-spiking fevers, evanescent skin rash, arthralgia/arthritis, neutrophilic leukocytosis and hyperferritinemia. In addition to these major manifestations, liver involvement is common but very heterogeneous, ranging from minimal liver enzyme elevation to life-threatening fulminant hepatic failure (3–7). The prevalence of elevated transaminases varies from 23 to 94%, according to different studies. However, a majority of them reported that more than half of the patients had abnormal liver function tests (LFTs) (3, 8–13). Although only limited data revealed the characteristics and outcomes of liver involvement in patients with AOSD, the detailed features of liver involvement remain rather scarce. For example, the time needed for recovery of the abnormal LFTs was ambiguous, and the relationship of LFTs with treatment and prognosis is undermined (3, 11).

The present study aims to illustrate the detailed information, treatment strategies and outcomes of liver involvement in patients with AOSD, to analyze the correlations of LFTs with other laboratory values and to further explore the prognostic importance.

## Materials and Methods

### Patients

A total of 128 AOSD patients admitted to the Department of Rheumatology and Immunology, Ruijin Hospital from July 2016 to August 2019 were consecutively enrolled and followed up. All patients met the Yamaguchi diagnostic criteria (14). Besides, patients with a history of alcohol abuse, evidence of other chronic hepatobiliary or pancreatic diseases were excluded. Informed consent was obtained from all patients, and the clinical records were anonymized before analysis. This survey was approved by the Institutional Research Ethics Committee of Ruijin Hospital (ID: 2016–62) and was conducted following the Principles of the Declaration of Helsinki.

### Data Collection

The demographic characteristics, comorbidities, clinical features, laboratory values, and treatment strategies were collected. The following clinical features were recorded: fever, typical rash, arthralgia, arthritis, myalgia, lymphadenopathy, sore throat, splenomegaly, hepatomegaly, abdominal pain, and sore throat. The splenomegaly, hepatomegaly and lymphadenopathy were evaluated by ultrasound or computed tomography (CT) scans. Pleural effusion or pleuritis and pneumonia were assessed by CT scans. Pericarditis was confirmed by echocardiography or CT scans.

The laboratory parameters recorded including complete blood counts, alanine aminotransferase (ALT), aspartate aminotransferase (AST), alkaline phosphatase (ALP), γ-glutamyl transpeptidase (GGT), prealbumin (preAlb), albumin (Alb), total bilirubin (TBil), prothrombin time (PT), lactate dehydrogenase (LDH), ferritin, C-reactive protein (CRP), erythrocyte sedimentation rate (ESR), levels of interleukin(IL)-1β, the soluble receptor of IL-2 (sIL-2R), IL-6, IL-8, IL-10, IL-18 and tumor necrosis factor (TNF)-α, and profiles of T-cell subsets (CD3^+^, CD4^+^, and CD8^+^) and B-cell subsets (CD19^+^ and CD20^+^). All laboratory tests were performed in a core laboratory.

The liver involvement was defined as hepatomegaly and/or elevation of any LFT throughout the disease course. The LFTs including ALT, AST, ALP, and GGT. The upper limits of normal (ULN) are 40 IU/L in ALT and AST, 126 IU/L in ALP, and 64 IU/L in GGT. For this study, the peak values of LFTs were recorded and the levels of LFTs elevation were categorized into normal, mild (higher than ULN but ≤2 ULN), moderate (higher than 2 ULN but ≤5 ULN) and severe (>5 ULN) according to times to ULN. Patients were grouped as normal, mild, moderate or severe LFT abnormality according to the highest level of elevation among the four LFTs. The LFTs were followed at various points during hospitalization and follow-up clinic visits to count the recovery time. However, the recovery time of three patients who died in the hospital was obtained.

The systemic score proposed by Pouchot et al. was calculated to evaluate the disease activity and severity (3). This scoring system counts the total number of the following 12 manifestations: fever, typical skin rash, sore throat, myalgia, abdominal pain, pneumonia, pleuritis, pericarditis, splenomegaly, lymphadenopathy, hepatomegaly or abnormal liver function tests, and leukocytes > 15,000/mm^3^. The diagnosis of hemophagocytic lymphohistiocytosis (HLH) was based on 2004-HLH criteria (15).

The treatment strategies were also recorded. The dosages of glucocorticoid were calculated equivalent to prednisolone, and a dose equivalent to prednisolone more than 100 mg was classified as a very high dose (16). Besides, refractory AOSD was defined as active disease status despite prednisolone over 1 mg/kg/day for more than 1 week with or without disease-modifying antirheumatic drugs (DMARDs). This was further confirmed by two experienced rheumatologists. The disease course of patients with AOSD was divided into three distinct types: monocyclic, polycyclic and chronic courses over more than 1 year of follow-up (1). The joint radiographs were obtained from patients with joint involvement. The disease pattern was considered a “chronic articular pattern” when patients had radiographic joint space narrowing, erosion, or ankylosis, otherwise, it was a systemic pattern (17).

### Statistics

Variables were presented as frequency counts (%) for categorical variables and median (interquartile range, IQR) for continuous data, while the values of LFTs were presented as median [range]. The continuous data were compared using Mann-Whitney *U*-test for two groups or Kruskal–Wallis tests for multiple groups. Proportions were analyzed using χ^2^ test or Fisher's exact test, as appropriate. Spearman correlation test was used to assess the correlations between LFTs with different variables. Receiver-operating characteristic (ROC) analyses were calculated to determine values at the maximum Youden index as cut-off points for continuous variables. Variables (including socio-demographic variables, clinical features, disease activity score and laboratory values) were further assessed by logistic regression analyses to estimate the risk for refractory AOSD. Variables identified in univariate analyses (*p* < 0.05) were then entered into a forward stepwise multivariable logistic regression model. All *p*-values were two-sided, and a *p* < 0.05 was considered statistically significant. All statistical analyses were performed with IBM SPSS Statistics for Mac, version 26.0 (IBM Corp., Armonk, N.Y., USA).

## Results

### Demographic Characteristics and Clinical Features of 128 Patients With AOSD

A total of 128 cases with AOSD were enrolled in the present study, and the characteristics of the participants are summarized in Table 1. The median age of the patients was 35 (27, 47) years old with a female predominance (79.7%). The most common clinical presentations were high fever (94.5%), arthralgia (93.8%), lymphadenopathy (89.8%), liver involvement (81.3%), and sore throat (80.5%). 77.3% of patients with AOSD had ferritin > 5 ULN and 61.7% had leukocytes > 15,000/mm^3^. The median systemic score was 7 (5, 8). We identified 104 (81.3%) patients with AOSD who had liver involvement, of which 18 (17.3%) had hepatomegaly. Besides, we found male patients tend to have liver involvement (*p* = 0.027), while no significant differences were observed in age, body mass index, or comorbidities between patients with and without liver involvement. Of note, patients with liver involvement had significantly higher systemic score (*p* < 0.001) and increased possibility of pleuritis (*p* = 0.023), pneumonia (*p* = 0.016), pericarditis (*p* = 0.001), and ferritin > 5 ULN (*p* = 0.027). Patients with liver involvement tended to exhibit a severer clinical picture with raised disease activity.

**Table 1:** Demographic and selected clinical features of AOSD patients.

**Variables**	**All patients**	**Patients with liver involvement**	**Patients without liver involvement**	***p-*values**
	***n* = 128**	***n* = 104**	***n* = 24**	
Age (years)	35 (27, 47)	35 (27, 47)	35 (28, 49)	0.903
Female	102 (79.7)	79 (76)	23 (95.8)	**0.027**
Body mass index (kg/m^2^)	21.47 (19.48, 23.31)	21.47 (19.40, 23.04)	21.59 (19.66, 23.57)	0.696
Diabetes mellitus	9 (7)	8 (7.7)	1 (4.2)	1.000
Hypertension	8 (6.3)	6 (5.8)	2 (8.3)	0.643
Disease duration (month)	2.3 (1.03, 10.04)	1.88 (0.92, 8.17)	4.46 (1.43, 18.37)	**0.035**
Fever > 39°C	121 (94.5)	98 (94.2)	23 (95.8)	1.000
Typical skin rash	57 (44.5)	44 (42.3)	13 (54.2)	0.292
Pleuritis	60 (46.9)	54 (51.9)	6 (25)	**0.017**
Pneumonia	44 (34.4)	41 (39.4)	3 (12.5)	**0.016**
Pericarditis	30 (23.4)	30 (28.8)	0 (0)	**0.001**
Myalgia	65 (50.8)	54 (51.9)	11 (45.8)	0.591
Splenomegaly	68 (53.1)	59 (56.7)	9 (37.5)	0.089
Hepatomegaly	18 (14.1)	18 (17.3)	0 (0)	**0.024**
Lymphadenopathy	115 (89.8)	92 (88.5)	23 (95.8)	0.460
Sore throat	103 (80.5)	82 (78.8)	21 (87.5)	0.406
Abdominal pain	12 (9.4)	11 (10.6)	1 (4.2)	0.462
Arthralgia	120 (93.8)	96 (92.3)	24 (100)	0.350
Arthritis	56 (43.8)	45 (43.3)	11 (45.8)	0.819
Leukocytes > 15,000/mm^3^	79 (61.7)	63 (60.6)	16 (66.7)	0.580
Ferritin > 5 ULN	99 (77.3)	85 (81.7)	14 (58.3)	**0.014**
Systemic score	7 (5, 8)	7 (6, 8)	5 (4.25, 6)	**<0.001**

*Data are presented as median (IQR) for continuous variables, and as frequency counts (%) for categorical variables. ULN, upper limit of normal. A p < 0.05 is shown in bold type*.

### The Liver Abnormalities and Recovery Time of LFTs in AOSD Patients With Liver Involvement

The features of liver abnormalities in patients with liver involvement were shown in Table 2. We found all patients with hepatomegaly had elevated LFTs. Among patients with liver involvement, 81.7% had elevated ALT, 87.5% had elevated AST, 51.0% had elevated ALP, and 72.1% had elevated GGT throughout the disease course. Furthermore, 78 (75.0%) patients had decreased preAlb, 88 (84.6%) patients had decreased Alb, 6 (5.8%) patients had elevated TBil, and 3 (2.9%) patients had prolonged PT. The median of peak ALT was 99 [11, 3,436] IU/L; the peak AST was 87 [16, 3,237] IU/L; the peak ALP was 132.5 [38, 491] IU/L; and the peak GGT was 117 [14, 696] IU/L. To evaluate the levels of elevation in patients with AOSD, we grouped the patients according to the highest levels of elevation among four LFTs and identified 34 (32.7%) patients with mild elevation, 32 (30.8%) patients with moderate elevation, and 38 (36.5%) patients with severe elevation.

**Table 2:** LFTs and associated laboratory values of patients with liver involvement.

**Variables**	***n* = 104**
Elevation of ALT	85 (81.7)
Elevation of AST	91 (87.5)
Elevation of ALP	53 (51.0)
Elevation of GGT	75 (72.1)
Peak ALT (IU/L)	99 [11, 3436]
Peak AST (IU/L)	87 [16, 3237]
Peak ALP (IU/L)	132.5 [38, 491]
Peak GGT (IU/L)	117 [14, 696]
Levels of elevation	
Mild (ULN < LFTs ≤ 2 ULN)	34 (32.7)
Moderate (2 ULN < LFTs ≤ 5 ULN)	32 (30.8)
Severe (LFTs > 5 ULN)	38 (36.5)
PreAlb < 180 mg/L	78 (75.0)
Alb < 35 g/L	88 (84.6)
TBil > 24 μmol/L	6 (5.8)
PT > 16 s	3 (2.9)
Hepatomegaly	18 (17.3)

*Data are presented as median [range] for the peak values of LFTs and as frequency counts (%) for categorical variables. LFTs, liver function tests; ALT, alanine aminotransferase; AST, aspartate aminotransferase; ALP, alkaline phosphatase; GGT, gamma-glutamyl transpeptidase; ULN, upper limits of normal; preAlb, prealbumin; Alb, albumin; TBil, total bilirubin; PT, prothrombin time*.

**Figure 1:**
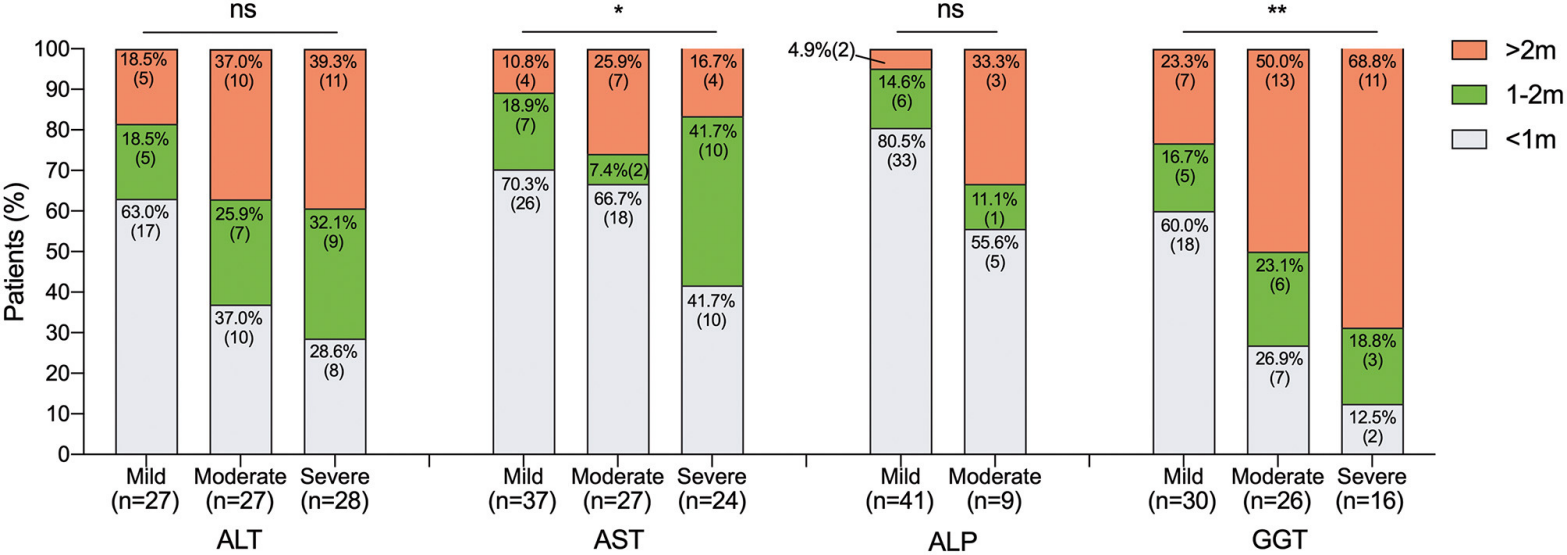
Recovery time of LFTs according to the levels of elevation. Data are expressed as % (*n*). Ns, not significant, *p < 0.05, and **p < 0.01. LFTs, liver function tests; ALT, alanine aminotransferase; AST, aspartate aminotransferase; ALP, alkaline phosphatase; GGT, gamma-glutamyl transpeptidase; m, month.

In addition, the distribution of LFTs recovery time was described in Figure 1 according to the levels of elevation, respectively. In general, the majority of elevated ALT, AST, and ALP decreased to normal within the range of 2 months, except for GGT. The mild elevated LFTs dropped to normal in a month were 63.0% in ALT, 70.3% in AST, 80.5% in ALP and 60.0% in GGT. Besides, 66.7% of the moderate elevated AST and 55.6% of moderate elevated ALP could recover in a month. The elevated AST seems to recover faster regardless of the levels of elevation. Only 16.7% of severe elevated AST recovered after 2 months. Conversely, it took more than 2 months for over one-third of moderate (37.0%), severe (39.3%) elevated ALT, and moderate (33.3%) elevated ALP, and at least half of moderate (50.0%) and severe (68.8%) elevated GGT took more than 2 months to recover. It appears that the recovery time was related to the levels of elevation. Typically, higher levels of elevated GGT had significantly longer recovery period (*p* = 0.009).

### The Correlations Between Baseline LFTs, Prealbumin and Albumin With Disease Activity Score and Laboratory Values

**Figure 2:**
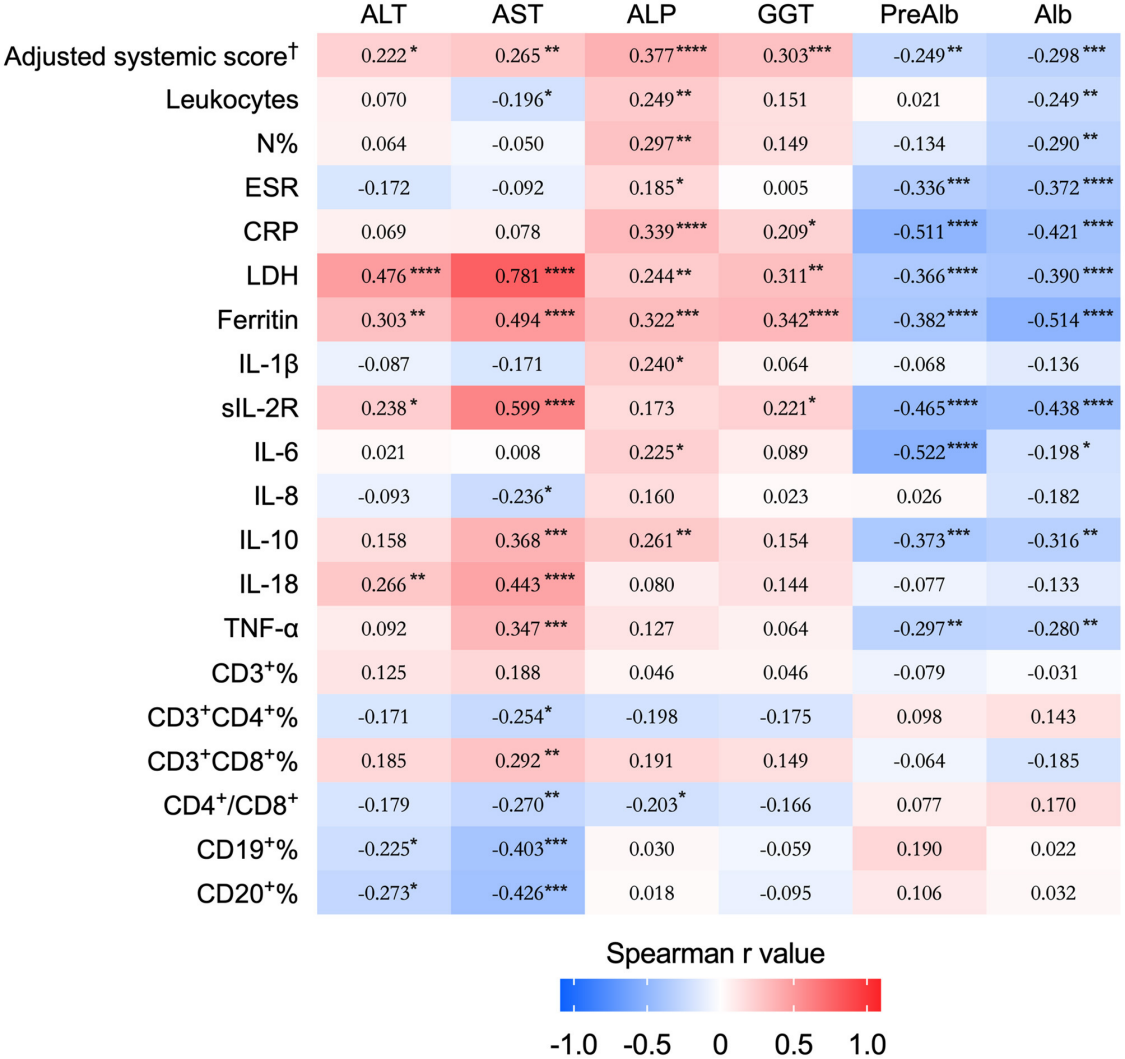
Correlation matrix of LFTs and prealbumin and albumin with disease activity score and laboratory parameters. The heat-map displays the Spearman's rank coefficient to show the correlation strength, and the significances are presented as *p < 0.05, **p < 0.01, ***p < 0.001, and ****p < 0.0001. ^†^The adjusted systemic score excluded the item “hepatomegaly or abnormal liver function” from the calculation of the score. LFTs, liver function tests; ALT, alanine aminotransferase; AST, aspartate aminotransferase; ALP, alkaline phosphatase; GGT, gamma-glutamyl transpeptidase; preAlb, prealbumin; Alb, albumin; N%, the percentage of neutrophils; ESR, erythrocyte sedimentation rate; CRP, C-reactive protein; LDH, lactate dehydrogenase; IL, interleukin; sIL-2R, soluble receptor of IL-2; TNF, tumor necrosis factor.

To investigate the relationship between liver function and disease activity, we conducted a correlation matrix based on Spearman r values between LFTs, preAlb and Alb with disease activity score as well as laboratory values (Figure 2). All LFTs were significantly positively correlated with the adjusted systemic score, LDH, and ferritin, while the preAlb and Alb were negatively correlated with the systemic score, leukocytes, N%, ESR, CRP, LDH, and ferritin. The AST had the highest correlation with the LDH (*r* = 0.781, *p* < 0.0001). Moreover, ALP was correlated with leukocytes, N%, CRP. Collectively, LFTs were associated with disease activity score and relevant laboratory tests.

In addition, we further explored the associations of LFTs, preAlb and Alb with IL-1β, sIL-2R, IL-6, IL-8, IL-10, and TNF-α (Figure 2). The AST was very highly correlated with sIL-2R (*r* = 0.599, *p* < 0.0001), followed by IL-18 (*r* = 0.443, *p* < 0.0001), IL-10 (*r* = 0.368, *p* = 0.0001), and TNF-α (*r* = 0.347, *p* = 0.0004). The ALT correlated with sIL-2R (*r* = 0.238, *p* = 0.015) and IL-18 (*r* = 0.266, *p* = 0.007). The preAlb significantly correlated with multiple cytokines including sIL-2R (*r* = −0.465, *p* < 0.0001), IL-6 (*r* = −0.522, *p* < 0.0001), IL-10 (*r* = −0.373, *p* = 0.0001), and TNF-α (*r* = −0.297, *p* = 0.0027), and the Alb was negatively correlated with sIL-2R (*r* = −0.438, *p* < 0.0001), IL-10 (*r* = −0.316, *p* = 0.0012) and TNF-α (*r* = −0.280, *p* = 0.0046).

To date, the expression of peripheral T and B cell subsets and the associations with LFTs were not elucidated. Interestingly, we found AST was positively correlated with the proportion of CD8+ T cells (*r* = 0.292, *p* = 0.004), and negatively correlated with CD4+ Tcells (*r* = −0.254, *p* = 0.013), and the percentage of CD19+ (*r* = −0.403, *p* = 0.0002) and CD20+ (*r* = −0.426, *p* = 0.0005) B cells.

### The Treatments and Outcomes of Patients According to the Levels of LFTs

To investigate the influence of LFTs on the treatments, the therapy strategies were compared among patients with different levels of LFTs (Table 3). Our analysis showed that patients with higher levels of elevation were ultimately prescribed with higher dosages of glucocorticoid (*p* < 0.001); moreover, 63.2% of patients with severe elevation required a very high dose of glucocorticoid (*p* < 0.001). With regards to DMARDs, patients with higher levels of LFTs were more often treated with cyclosporine A (CsA) (*p* = 0.015), intravenous immunoglobulin (IVIG) (*p* < 0.001) and etoposide (*p* < 0.001), while less likely to be treated with methotrexate (MTX) (*p* < 0.001) and hydroxychloroquine (HCQ) (*p* = 0.014). There was no significant difference in the application of biologics. Among all receiving biologic agents, one patient with a severe elevation of LFTs was refractory to multiple therapeutic options and was finally treated with glucocorticoid combined with tofacitinib plus anakinra during follow-up. As a result, patients with high levels of LFTs tend to require more intensive treatments.

**Table 3:** The treatments and outcomes of patients according to the levels of LFTs elevation.

	**Normal (LFTs ≤ ULN)**	**Mild (ULN < LFTs ≤ 2 ULN)**	**Moderate (2 ULN < LFTs ≤ 5 ULN)**	**Severe (LFTs > 5 ULN)**	***p*-values**
	***n* = 24**	***n* = 34**	***n* = 32**	***n* = 38**	
Treatments					
Application of glucocorticoid	22 (91.7)	33 (97.1)	32 (100)	37 (97.4)	0.401
Dosage (mg)^†^	75 (26.25, 100)	100 (50, 108.13)	100 (56.25, 145.81)	175 (100, 200)	**<0.001**
Very high dose of glucocorticoid^‡^	2 (8.3)	8 (23.5)	9 (28.1)	24 (63.2)	**<0.001**
Application of MTX	19 (79.2)	26 (76.5)	21 (65.6)	12 (31.6)	**<0.001**
Application of CsA	5 (20.8)	7 (20.6)	5 (15.6)	18 (47.4)	**0.015**
Application of HCQ	18 (75)	23 (67.6)	15 (46.9)	15 (39.5)	**0.014**
Application of biologic agents	5 (20.8)	4 (11.8)	6 (18.8)	3 (7.9)^§^	0.404
Tocilizumab	1 (4.2)	1 (2.9)	1 (3.1)	1 (2.6)	
Etanercept	1 (4.2)	1 (2.9)	1 (3.1)	0	
Tofacitinib	3 (12.5)	2 (5.9)	4 (12.5)	2 (5.3)	
Anakinra	0	0	0	1 (2.6)	
Application of IVIG	0	1 (2.9)	3 (9.4)	15 (39.5)	**<0.001**
Application of etoposide	0	0	2 (6.3)	10 (26.3)	**<0.001**
Outcomes					
Disease pattern					
Systemic pattern	22 (91.7)	30 (88.2)	27 (84.4)	35 (92.1)	0.776
Chronic articular pattern	2 (8.3)	4 (11.8)	5 (15.6)	3 (7.9)	
Disease course					
Monocyclic course	12 (50)	24 (70.6)	15 (46.9)	22 (57.9)	0.164
Polycyclic course	7 (29.2)	6 (17.6)	5 (15.6)	8 (21.1)	
Chronic course	5 (20.8)	4 (11.8)	12 (37.5)	5 (13.2)	
Refractory AOSD	6 (25)	11 (32.4)	13 (40.6)	26 (68.4)	**0.001**
HLH	0	2 (5.9)	4 (12.5)	17 (44.7)	**<0.001**
Death	0	0	0	3 (7.9)	0.063

† Equivalent to prednisolone;

‡ Dosage equivalent to prednisolone > 100 mg was considered very high dose of glucocorticoid;

§ *One patient was prescribed with tofacitinib plus anakinra. Data are presented as median (IQR) for continuous variables, and as frequency counts (%) for categorical variables. A p < 0.05 is shown in bold type. LFTs, liver function tests; ULN, upper limits of normal; MTX, methotrexate; CsA, cyclosporine A; HCQ, hydroxychloroquine; IVIG, intravenous immunoglobulin; HLH, hemophagocytic lymphohistiocytosis*.

Besides, our results showed that the incidence of HLH (*p* < 0.001) was significantly higher in patients with higher levels of LFTs elevation, the same as the incidence of refractory AOSD (*p* = 0.001). Furthermore, three in-hospital died patients had significantly elevated LFTs with peak ALT 787 [740, 1932] IU/L, AST 3102 [1435, 3237] IU/L, ALP 318 [218, 341] IU/L, and GGT 201 [133, 273] IU/L, and two of them suffered from acute hepatic failure. However, the disease courses and patterns were similar among different levels of LFTs elevation over more than 1-year follow-up.

### The Predictive Values of the LFTs in Refractory AOSD

The response to therapy varied in patients with AOSD and was difficult to predict. As shown in Table 3, we found patients with significant liver involvement tend to be refractory AOSD. To evaluate the predictive values of the clinical and laboratory variables in refractory AOSD, we further performed logistic regression analyses (Table 4). The univariate analyses indicated that these factors significantly associated with refractory AOSD: fever, skin rash, arthritis, splenomegaly, hepatomegaly, pleuritis, pneumonia, ferritin ≥ 3,427 ng/ml, ESR ≥ 69 mm/h, CRP ≥ 127.5 mg/L, N% ≥ 86.3%, Hb ≤ 109.5 g/L, LDH ≥ 450 IU/L, ALT ≥ 87 IU/L, AST ≥ 111 IU/L, ALP ≥ 141 IU/L, GGT ≥ 132 IU/L, preAlb ≤ 163 mg/L, Alb ≤ 31.5 g/L or PT ≥ 13.55s. Given the association between many of the covariates, multiple logistic regression analysis was then performed, simultaneously including all variables with statistical significance. Multivariable logistic regression analysis by stepwise forward selection identified the skin rash (OR: 5.66; 95%CI: 1.06, 30.11), splenomegaly (OR: 5.27; 95%CI: 1.77, 15.67), ESR ≥ 69 mm/h (OR: 6.95; 95%CI: 2.17, 22.21), ALP ≥ 141 IU/L (OR: 5.48; 95%CI: 1.40, 21.55) and GGT ≥ 132 IU/L (OR: 5.13; 95%CI: 1.30, 20.22) as independent predictors of refractory AOSD. Thus, the LFTs may be predictors for refractory AOSD, especially ALP and GGT, which are independent risk factors.

**Table 4:** Logistic regression of risk factors of refractory AOSD.

**Parameter^†^**	**Univariate analysis**		**Multivariate analysis**	
	**OR (95%CI)**	***p*-value**	**OR (95%CI)**	***p*-value**
Fever	4.35 (1.91, 9.93)	**<0.001**		
Skin rash	3.43 (1.28, 9.21)	**0.015**	5.66 (1.06, 30.11)	**0.042**
Arthritis	2.44 (1.13, 5.27)	**0.033**		
Splenomegaly	5.00 (2.34, 10.68)	**<0.001**	5.27 (1.77, 15.67)	**0.003**
Hepatomegaly	9.55 (2.04, 44.69)	**0.001**		
Pleuritis	4.02 (1.91, 8.46)	**<0.001**		
Pneumonia	2.65 (1.22, 5.77)	**0.019**		
Ferritin ≥ 3,427 (ng/mL)	4.58 (1.95, 10.73)	**<0.001**		
ESR ≥ 69 (mm/h)	3.31 (1.54, 7.13)	**0.002**	6.95 (2.17, 22.21)	**0.001**
CRP ≥ 127.5 (mg/L)	3.05 (1.24, 7.51)	**0.015**		
N% ≥ 86.3%	2.70 (1.15, 6.34)	**0.032**		
Hb ≤ 109.5 (g/L)	3.40 (1.62, 7.12)	**0.001**		
LDH ≥ 450 (IU/L)	3.80 (1.77, 8.17)	**0.001**		
ALT ≥ 87 (IU/L)	4.88 (2.20, 10.82)	**<0.001**		
AST ≥ 111 (IU/L)	6.00 (2.43, 14.84)	**<0.001**		
ALP ≥ 141 (IU/L)	8.27 (3.53, 19.38)	**<0.001**	5.48 (1.40, 21.55)	**0.015**
GGT ≥ 132 (IU/L)	8.00 (3.24, 19.73)	**<0.001**	5.13 (1.30, 20.22)	**0.020**
PreAlb ≤ 163 (mg/L)	2.50 (1.13, 5.55)	**0.034**		
Alb ≤ 31.5 (g/L)	2.96 (1.37, 6.42)	**0.006**		
PT ≥ 13.55 (s)	3.50 (1.43, 8.58)	**0.005**		

† *ROC analyses were applied to determine values at the maximum Youden index as cut-off points for continuous variables. Data are presented as OR (95%CI). A p < 0.05 is shown in bold type. ESR, erythrocyte sedimentation rate; CRP, C-reactive protein; N%, the percentage of neutrophils; Hb, hemoglobin; LDH, lactate dehydrogenase; ALT, alanine aminotransferase; AST, aspartate aminotransferase; ALP, alkaline phosphatase; GGT, gamma-glutamyl transpeptidase; preAlb, prealbumin; Alb, albumin; PT, prothrombin time; OR, odds ratio; CI, confidence interval*.

## Discussion

AOSD is a systemic inflammatory disease, and liver involvement is frequently observed but quite heterogeneous with non-specific histopathologic changes (3). However, the characteristics and prognosis of liver involvement remain not well-elucidated. Herein, we first described the detailed data of LFTs in patients with AOSD, analyzed the associations with disease activity score and laboratory values, represented the distribution of recovery time, and demonstrated the prognostic importance of LFTs.

The presence of liver involvement varies in different countries and races (3, 8–11, 13). Zhu et al. found that 62.3% of the patients with AOSD had abnormal transaminases, 32.9% had elevated ALP, and 48.1% had elevated GGT (11). The higher incidence of abnormal LFTs in our cohort may related to the longer observation period and the enrolment of only hospitalized patients. Besides, the liver abnormalities in patients with AOSD were considered mainly a mild to moderate increase in aminotransferase activity (1). Zhu et al. revealed one-quarter of the patients with abnormal transaminases were five times higher than ULN (11). Consistently, the elevation of LFTs was mild to moderate in more than half of the patients, while more than one-third of the patients had severe LFTs elevation. The abnormal liver enzymes are usually mild cytolysis, but severe cytolysis and cholestasis may occur (3, 11). Formerly, the elevation of liver enzymes was reported mostly transient (3, 11, 18). Our study, for the first time, described the distribution of recovery time of abnormal LFTs. However, the recovery time was not as quick as expected, especially in GGT. In general, liver involvement is very common and, in most cases, not severe. But high levels of LFTs could occur in some patients so that close monitoring would be needed.

The correlation analyses of LFTs with disease biomarkers showed a significant correlation with LDH, an indicator of cell death and tissue damage (19). Besides, strong associations were found with ferritin, high levels of which act as a sign of macrophage activation and have been hypothesized as a pathogenic protein contributing to the development of a self-perpetuating cytokine storm (20, 21). The cytokine cascade plays an important role in the pathogenesis of AOSD (1). Regarding the cytokines, we found that AST had the strongest correlation with sIL-2R, a truncated protein cleaved from the IL-2Rα protein when T cells are activated, acting as a surrogate indicator of T cell activation as well as an important diagnostic marker of HLH, followed by IL-18, IL-10, and TNF-α, cytokines reflecting the diseases activity of AOSD (22–24). Previously study showed that IL-18 markedly increased in AOSD patients, and Priori et al. revealed intensive expression of macrophage-derived IL-18 in liver parenchyma in an AOSD patient with hepatitis, which indicated that IL-18 may contribute to liver damage (6, 25, 26).

The adaptive immunity is also considered involved in the pathogenesis of AOSD including deficiency in regulatory T cells (26). We found the percentage of CD8^+^ T cells positively associated with AST, while the CD4^+^/CD8^+^ ratio, the rates of CD19^+^ and CD20^+^ subsets of B cells correlated negatively. Jung et al. further explored the T cell differentiation in 14 patients with AOSD. They demonstrated that ALT and AST positively correlated with CD4^+^ naïve T cells and CD4^+^ central memory T cells, and negatively correlated with CD4^+^ effector memory T cells, and ALT positively correlated with CD8^+^ central memory T cells (27). However, the data of the B-cell subsets were scarce, although a successful treatment of refractory AOSD with B cell depletion was reported (28). A thorough profile of peripheral immunophenotyping in patients with AOSD is required to investigate further.

In addition, we first discovered that the levels of preAlb and Alb in patients with AOSD were negatively associated with multiple well-known disease activity biomarkers, which reflect disease activity. Although the reduced levels of preAlb and Alb might result from malnutrition, the systemic inflammation can also suppress the production of preAlb and Alb, as part of the acute phase response (29–32). The decreased levels of these two proteins were also described in rheumatoid arthritis (RA), and the Alb levels were found to reflect disease activity (33, 34). Besides, the serum preAlb or Alb levels were previously revealed associated with poor prognosis in systemic sclerosis, cardiovascular diseases and renal diseases (35–38). In conclusion, the levels of preAlb and Alb are also disease activity markers in patients with AOSD.

Besides, we revealed that patients with higher levels of LFTs had a higher possibility of developing HLH, which is a life-threatening complication of AOSD (39). According to Ruscitti et al., patients with liver involvement had an almost 6-fold higher risk of HLH than those without (39). Néel et al. observed the association of bone marrow hemophagocytosis with AST in AOSD (40). Patients with high levels of LFTs might indicate exaggerated inflammatory responses requiring a high dose of glucocorticoids and/or more powerful immunosuppressants. As secondary HLH is commonly triggered by infections, malignancies, or autoinflammatory/autoimmune disorders, we think that liver function abnormalities should weight more for the diagnose of HLH secondary to AOSD, as it is already included in the recent criteria for MAS in systemic juvenile idiopathic arthritis (sJIA), a continuum of a single disease entity of AOSD (41).

However, it's quite a management dilemma that patients with active AOSD with a severe elevation of LFTs required more intensive therapies, but hepatic toxicity was reported in some drugs (42–44). Although a previous study showed that the presence of LFTs abnormalities does not contraindicate methotrexate prescription, the administration of MTX and HCQ declined with the elevation of LFTs in our cohort (45). Néel et al. showed that the efficacy rate of IVIGs, cyclosporine, and anakinra were 11/27 (41%), 13/18 (72%), and 8/9 (89%), respectively in patients with AOSD admitted to intensive care medicine (ICU) (40). From our results, the application rate of CsA, etoposide, IVIGs and a high dose of glucocorticoids was higher in patients with severe elevation of LFTs. To sum up, the patients with higher levels of LFTs may be more severe and ultimately required more intensive treatments in clinical practice.

The combination of heterogeneous symptoms, complex laboratory results and polymorphic prognosis predicts responses to treatment extremely difficult in patients with AOSD. Néel et al. found that the overall response rate of glucocorticoids was only 50% (40). A similar response rate was identified in our cohort; in addition, we found that refractory AOSD was more common in patients with a higher level of LFTs. However, a predictive tool for refractory AOSD was not available yet. Our analyses showed that multiple factors could reflect the risk of refractoriness; more importantly, two of the LFTs (ALP ≥ 141 IU/L and GGT ≥ 132 IU/L) were independent predictive factors for refractory AOSD. Several newly biologic agents, such as anakinra, tocilizumab and tofacitinib, showed strong efficacy and steroid-sparing effects in AOSD patients (46–49). As a result, the prediction of refractory AOSD may be helpful in timely tailoring therapy to improve the prognosis.

Our study provided details of liver involvement in AOSD patients with relatively large sample size. We revealed a high proportion of patients with elevated LFTs and recorded the recovery time of liver enzymes. Also, we analyzed the association of LFTs with detail clinical features and various cytokines, treatment strategies and prognosis. Furthermore, we explored the risk factors that contributed to refractory AOSD and proposed potential predictive factors for the first time. However, there are still some limitations. Firstly, due to the retrospective design setting, it's hard to completely exclude the effects of confounding factors such as treatments. Besides, this study was based on hospitalized patients in a tertiary hospital which might lead to selection bias. As a result, multi-center well-designed studies are needed to verify the results of our cohort.

## Conclusion

To sum up, our study confirmed that the liver involvement was common; meanwhile, the elevated liver enzymes correlated with disease activity in patients with AOSD and the recovery time of abnormal LFTs was not always as quick as we expected. Besides, patients with higher levels of LFTs tend to receive more intensive treatments and suffer from poorer prognosis. Lastly, several biochemical biomarkers could be predictors of refractory AOSD, especially elevated ALP and GGT, which are independent risk factors.

## Data Availability Statement

The raw data supporting the conclusions of this article will be made available by the authors, without undue reservation.

## Ethics Statement

The studies involving human participants were reviewed and approved by The Institutional Research Ethics Committee of Ruijin Hospital, Shanghai Jiao Tong University School of Medicine. The patients/participants provided their written informed consent to participate in this study.

## Author Contributions

YS (25th author), CY, HC, and ZW: study conception and design. HC, JM, PH, LZ, YS (8th author), QH, HZ, LW, HL, XC (12th author), JY, HS, XW, JJ, TL, ZZ, XQ, MW, FW, and XC (23rd author): acquisition of data. HC, YS (25th author), and CY: drafting and revising the article. HC, TF, and JT: analysis and interpretation of data. All authors reviewed this article and approved the final manuscript.

## Conflict of Interest

The authors declare that the research was conducted in the absence of any commercial or financial relationships that could be construed as a potential conflict of interest.

## References

[1] Feist E Mitrovic S Fautrel B Mechanisms, biomarkers and targets for adult-onset Still's disease. Nat Rev Rheumatol. (2018) 14:603–18. 10.1038/s41584-018-0081-x 30218025 PMC7097309

[2] Wang M-Y Jia J-C Yang C-D Hu Q-Y. Pathogenesis, disease course, and prognosis of adult-onset Still's disease. Chin Med J. (2019) 132:2856–64. 10.1097/CM90000000000000538 31856058 PMC6940076

[3] Pouchot J Sampalis JS Beaudet F Carette S Decary F Salusinsky-Sternbach M. Adult Still's disease: manifestations, disease course, and outcome in 62 patients. Med (Baltimore). (1991) 70:118–36. 10.1097/00005792-199103000-00004 2005777

[4] Dino O Provenzano G Giannuoli G Sciarrino E Pouyet M Pagliaro L. Fulminant hepatic failure in adult onset Still's disease. J Rheumatol. (1996) 23:784–5. 8730149

[5] Taccone FS Lucidi V Donckier V Bourgeois N Decaux G Vandergheynst F. Fulminant hepatitis requiring MARS and liver transplantation in a patient with Still's disease. Eur J Intern Med. (2008) 19:e26–8. 10.1016/j.ejim.2007.06025 18848162

[6] Ogata A Kitano M Yamanaka J Yamasaki T Hashimoto N Iwasaki T. Interleukin 18 and hepatocyte growth factor in fulminant hepatic failure of adult onset Still's disease. J Rheumatol. (2003) 30:1093–6. 12734913

[7] Nagashima T Aoki Y Onishi S Iwamoto M Okazaki H Minota S. Steroid-refractory severe hepatic failure in adult onset Still's disease responding to cyclosporine. Clin Rheumatol. (2008) 27:1451–3. 10.1007/s10067-008-0950-9 18592135

[8] Ohta A Yamaguchi M Tsunematsu T Kasukawa R Mizushima H Kashiwagi H. Adult Still's disease: a multicenter survey of Japanese patients. J Rheumatol. (1990) 17:1058–63. 2213780

[9] Mok CC Lau CS Wong RW. Clinical characteristics, treatment, and outcome of adult onset Still's disease in southern Chinese. J Rheumatol. (1998) 25:2345–51. 9858428

[10] Zeng T Zou YQ Wu MF Yang CD. Clinical features and prognosis of adult-onset Still's disease: 61 cases from China. J Rheumatol. (2009) 36:1026–31. 10.3899/jrheum080365 19273456

[11] Zhu G Liu G Liu Y Xie Q Shi G Liver abnormalities in adult onset Still's disease: a retrospective study of 77 Chinese patients. J Clin Rheumatol. (2009) 15:284–8. 10.1097/RHU0b013e3181b57199 19734733

[12] Kalyoncu U Solmaz D Emmungil H Yazici A Kasifoglu T Kimyon G. Response rate of initial conventional treatments, disease course, and related factors of patients with adult-onset Still's disease: Data from a large multicenter cohort. J Autoimmun. (2016) 69:59–63. 10.1016/j.jaut.2016.02010 26970681

[13] Hu QY Zeng T Sun CY Luo CN Liu S Ding TT. Clinical features and current treatments of adult-onset Still's disease: a multicentre survey of 517 patients in China. Clin Exp Rheumatol. (2019) 37(Suppl. 121):52–7. 31573475

[14] Yamaguchi M Ohta A Tsunematsu T Kasukawa R Mizushima Y Kashiwagi H. Preliminary criteria for classification of adult Still's disease. J Rheumatol. (1992) 19:424–30. 1578458

[15] Henter JI Horne A Arico M Egeler RM Filipovich AH Imashuku S. HLH-2004: diagnostic and therapeutic guidelines for hemophagocytic lymphohistiocytosis. Pediatr Blood Cancer. (2007) 48:124–31. 10.1002/pbc21039 16937360

[16] Buttgereit F da Silva JA Boers M Burmester GR Cutolo M Jacobs J. Standardised nomenclature for glucocorticoid dosages and glucocorticoid treatment regimens: current questions and tentative answers in rheumatology. Ann Rheum Dis. (2002) 61:718–22. 10.1136/ard.61.8718 12117678 PMC1754188

[17] Franchini S Dagna L Salvo F Aiello P Baldissera E Sabbadini MG. Efficacy of traditional and biologic agents in different clinical phenotypes of adult-onset Still's disease. Arthritis Rheum. (2010) 62:2530–5. 10.1002/art27532 20506370

[18] Andres E Kurtz JE Perrin AE Pflumio F Ruellan A Goichot B. Retrospective monocentric study of 17 patients with adult Still's disease, with special focus on liver abnormalities. Hepatogastroenterology. (2003) 50:192–5. 12630021

[19] Drent M Cobben NA Henderson RF Wouters EF van Dieijen-Visser M Usefulness of lactate dehydrogenase and its isoenzymes as indicators of lung damage or inflammation. Eur Respir J. (1996) 9:1736–42. 10.1183/09031936.9609081736 8866602

[20] Ruddell RG Hoang-Le D Barwood JM Rutherford PS Piva TJ Watters DJ. Ferritin functions as a proinflammatory cytokine via iron-independent protein kinase C zeta/nuclear factor kappaB-regulated signaling in rat hepatic stellate cells. Hepatology. (2009) 49:887–900. 10.1002/hep22716 19241483 PMC2701483

[21] Rosario C Zandman-Goddard G Meyron-Holtz EG D'Cruz DP Shoenfeld Y. The hyperferritinemic syndrome: macrophage activation syndrome, Still's disease, septic shock and catastrophic antiphospholipid syndrome. BMC Med. (2013) 11:185. 10.1186/1741-7015-11-185 23968282 PMC3751883

[22] Coca A Bundy KW Marston B Huggins J Looney RJ. Macrophage activation syndrome: serological markers and treatment with anti-thymocyte globulin. Clin Immunol. (2009) 132:10–8. 10.1016/j.clim.2009.02005 19297252

[23] Lin M Park S Hayden A Giustini D Trinkaus M Pudek M. Clinical utility of soluble interleukin-2 receptor in hemophagocytic syndromes: a systematic scoping review. Ann Hematol. (2017) 96:1241–51. 10.1007/s00277-017-2993-y 28497365

[24] Sun Y Wang Z Chi H Hu Q Ye J Liu H. Elevated serum levels of interleukin-10 in adult-onset Still's disease are associated with disease activity. Clin Rheumatol. (2019) 38:3205–10. 10.1007/s10067-019-04642-x 31236746

[25] Priori R Barone F Alessandri C Colafrancesco S McInnes IB Pitzalis C. Markedly increased IL-18 liver expression in adult-onset Still's disease-related hepatitis. Rheumatology (Oxford). (2011) 50:776–80. 10.1093/rheumatology/keq397 21149398

[26] Priori R Colafrancesco S Alessandri C Minniti A Perricone C Iaiani G. Interleukin 18: a biomarker for differential diagnosis between adult-onset Still's disease and sepsis. J Rheumatol. (2014) 41:1118–23. 10.3899/jrheum130575 24786926

[27] Kim HA Jung JY Suh CH Sohn S. Characteristic patterns of HLA presentation and T cell differentiation in adult-onset Still's disease. Ann Rheum Dis. (2018) 77:1216. 10.1136/annrheumdis-2018-eular6246 30052100 PMC6073833

[28] Ahmadi-Simab K Lamprecht P Jankowiak C Gross WL. Successful treatment of refractory adult onset Still's disease with rituximab. Ann Rheum Dis. (2006) 65:1117–8. 10.1136/ard.2005047621 16837497 PMC1798247

[29] Gabay C Kushner I. Acute-phase proteins and other systemic responses to inflammation. N Engl J Med. (1999) 340:448–54. 10.1056/NEJM199902113400607 9971870

[30] Fuhrman MP Charney P Mueller CM. Hepatic proteins and nutrition assessment. J Am Diet Assoc. (2004) 104:1258–64. 10.1016/j.jada.2004.05213 15281044

[31] Eckart A Struja T Kutz A Baumgartner A Baumgartner T Zurfluh S. Relationship of nutritional status, inflammation, and serum albumin levels during acute illness: a prospective study. Am J Med. (2019) 133:713–22. 10.1016/j.amjmed.2019.10.031 31751531

[32] Dickson PW Howlett GJ Schreiber G. Metabolism of prealbumin in rats and changes induced by acute inflammation. Eur J Biochem. (1982) 129:289–93. 10.1111/j.1432-1033.1982.tb07051x 7151801

[33] Surrall KE Bird HA Dixon JS. Caeruloplasmin, prealbumin and alpha 2-macroglobulin as potential indices of disease activity in different arthritides. Clin Rheumatol. (1987) 6:64–9. 10.1007/BF02201003 2438080

[34] Hayashi H Satoi K Sato-Mito N Kaburagi T Yoshino H Higaki M. Nutritional status in relation to adipokines and oxidative stress is associated with disease activity in patients with rheumatoid arthritis. Nutrition. (2012) 28:1109–14. 10.1016/j.nut.2012.02009 23044162

[35] Codullo V Cereda E Klersy C Cavazzana I Alpini C Bonardi C. Serum prealbumin is an independent predictor of mortality in systemic sclerosis outpatients. Rheumatology (Oxford). (2016) 55:315–19. 10.1093/rheumatology/kev322 26359329

[36] Arques S. Human serum albumin in cardiovascular diseases. Eur J Intern Med. (2018) 52:8–12. 10.1016/j.ejim.2018.04014 29680174

[37] Sun J Axelsson J Machowska A Heimburger O Barany P Lindholm B Biomarkers of cardiovascular disease and mortality risk in patients with advanced CKD. Clin J Am Soc Nephrol. (2016) 11:1163–72. 10.2215/CJN10441015 27281698 PMC4934843

[38] Chertow GM Ackert K Lew NL Lazarus JM Lowrie EG. Prealbumin is as important as albumin in the nutritional assessment of hemodialysis patients. Kidney Int. (2000) 58:2512–7. 10.1046/j.1523-1755.2000.00435x 11115085

[39] Ruscitti P Iacono D Ciccia F Emmi G Cipriani P Grembiale RD. Macrophage activation syndrome in patients affected by adult-onset still disease: analysis of survival rates and predictive factors in the gruppo Italiano di ricerca in reumatologia clinica e sperimentale cohort. J Rheumatol. (2018) 45:864–72. 10.3899/jrheum170955 29657144

[40] Néel A Wahbi A Tessoulin B Boileau J Carpentier D Decaux O. Diagnostic and management of life-threatening Adult-Onset Still Disease: a French nationwide multicenter study and systematic literature review. Crit Care. (2018) 22:88. 10.1186/s13054-018-2012-2 29642928 PMC5896069

[41] Minoia F Bovis F Davi S Horne A Fischbach M Frosch M. Development and initial validation of the MS score for diagnosis of macrophage activation syndrome in systemic juvenile idiopathic arthritis. Ann Rheum Dis. (2019) 78:1357–62. 10.1136/annrheumdis-2019-215211 31296501

[42] Makin AJ Wendon J Fitt S Portmann BC Williams R. Fulminant hepatic failure secondary to hydroxychloroquine. Gut. (1994) 35:569–70. 10.1136/gut.35.4569 8175002 PMC1374814

[43] Kivity S Zafrir Y Loebstein R Pauzner R Mouallem M Mayan H. Clinical characteristics and risk factors for low dose methotrexate toxicity: a cohort of 28 patients. Autoimmun Rev. (2014) 13:1109–13. 10.1016/j.autrev.2014.08027 25172240

[44] Sepriano A Kerschbaumer A Smolen JS van der Heijde D Dougados M van Vollenhoven R. Safety of synthetic and biological DMARDs: a systematic literature review informing the 2019 update of the EULAR recommendations for the management of rheumatoid arthritis. Ann Rheum Dis. (2020) 79:760–70. 10.1136/annrheumdis-2019-216653 32033941

[45] Fujii T Akizuki M Kameda H Matsumura M Hirakata M Yoshida T. Methotrexate treatment in patients with adult onset Still's disease–retrospective study of 13 Japanese cases. Ann Rheum Dis. (1997) 56:144–48. 10.1136/ard.56.2144 9068291 PMC1752327

[46] Colafrancesco S Priori R Valesini G Argolini L Baldissera E Bartoloni E. Response to interleukin-1 inhibitors in 140 Italian patients with Adult-Onset Still's Disease: a multicentre retrospective observational study. Front Pharmacol. (2017) 8:369. 10.3389/fphar.201700369 28659802 PMC5469286

[47] Gabay C Fautrel B Rech J Spertini F Feist E Kotter I. Open-label, multicentre, dose-escalating phase II clinical trial on the safety and efficacy of tadekinig alfa (IL-18BP) in adult-onset Still's disease. Ann Rheum Dis. (2018) 77:840–7. 10.1136/annrheumdis-2017-212608 29472362 PMC5965361

[48] Kaneko Y Kameda H Ikeda K Ishii T Murakami K Takamatsu H. Tocilizumab in patients with adult-onset Still's disease refractory to glucocorticoid treatment: a randomised, double-blind, placebo-controlled phase III trial. Ann Rheum Dis. (2018) 77:1720–9. 10.1136/annrheumdis-2018-213920 30279267

[49] Hu Q Wang M Jia J Teng J Chi H Liu T. Tofacitinib in refractory adult-onset Still's disease: 14 cases from a single centre in China. Ann Rheum Dis. (2020) 79:842–4. 10.1136/annrheumdis-2019-216699 32079571 PMC7286046

